# Review of Studies on Older Drivers’ Behavior and Stress—Methods, Results, and Outlook

**DOI:** 10.3390/s21103503

**Published:** 2021-05-18

**Authors:** Yanning Zhao, Toshiyuki Yamamoto

**Affiliations:** 1 Department of Civil Engineering, Nagoya University, Nagoya 464-8601, Japan; 2 Institute of Materials and Systems for Sustainability, Nagoya University, Nagoya 464-8603, Japan; yamamoto@civil.nagoya-u.ac.jp

**Keywords:** older driver, biomedical signals, driving behavior, driving stress

## Abstract

This paper presents a review on relevant studies and reports related to older drivers’ behavior and stress. Questionnaires, simulators, and on-road/in-vehicle systems are used to collect driving data in most studies. In addition, research either directly compares older drivers and the other drivers or considers participants according to various age groups. Nevertheless, the definition of ‘older driver’ varies not only across studies but also across different government reports. Although questionnaire surveys are widely used to affordably obtain massive data in a short time, they lack objectivity. In contrast, biomedical information can increase the reliability of a driving stress assessment when collected in environments such as driving simulators and on-road experiments. Various studies determined that driving behavior and stress remain stable regardless of age, whereas others reported degradation of driving abilities and increased driving stress among older drivers. Instead of age, many researchers recommended considering other influencing factors, such as gender, living area, and driving experience. To mitigate bias in findings, this literature review suggests a hybrid method by applying surveys and collecting on-road/in-vehicle data.

## 1. Introduction

With global population ageing, more and more academics and governments are concerned with older drivers’ behavior, psychology, and driving safety [1,2,3]. Given its high proportion of older adults, Japan will have an estimated 25% more older drivers by 2030, increasing from 16 million in 2015 to 20 million in 2030 [4]. According to various official reports in the USA [5,6,7], the rate of older drivers has increased faster than that of any other age group, and the population over 70 years old is projected to increase by more than 70% between 2014 and 2030. Moreover, in 2013, the number of driving license holders over 70 years old in the UK surpassed 4 million (4,018,900) for the first time, and the rate of elderly drivers in the European Union will exceed one-quarter of the total by 2050 [8].

Given this trend, road traffic incidents involving older drivers represent a worldwide concern. The Older Drivers Task Force, a UK group of experts from a wide range of disciplines with an interest in the safety of older drivers, pointed that” this influx of older drivers has important economic and social value but it also presents road safety risks if we don’t adapt” [9]. Considering Japanese statistics, road safety in Tokyo is improving considerably, and the number of collisions has reduced by more than 50% since 2005. However, the ratio of older drivers involved in road traffic incidents has nearly doubled over the same time frame (from 10.9% to 20.4%) [4]. In the USA, 16 older adults were killed and 648 were injured in crashes, on an average, every day of 2014 [10,11]. The UK and Australia are facing the same problem, as statistics show that older drivers have the highest risk of death during road traffic incidents than drivers in any other age group in both countries [12,13].

Although reports suggest that the driving safety of older drivers is a serious global problem, its direct relation to older drivers’ physical and mental changes remains debatable.

Langford et al. [14] reached two findings: long periods of driving experience reduce the number of crashes per kilometer, and older adults drive less time than others. Hence, the relatively high crash rate of older drivers might be partially associated to their reduced driving periods. In this study, data from the Netherlands were analyzed to obtain the crash rates according to the yearly driving distance. However, the authors confirmed that most older adults (above 75 years old) drive safer than individuals in any other age group. Similarly, Mitchell [15] conducted a case study in Britain and concluded that the increase of fatality rates for older drivers may be caused by their fragility. Fildes et al. [16] confirmed that older road users are much more likely to be severely injured in crashes than their younger counterparts. A report by the Older Drivers Task Force in the UK [9] reached to a similar conclusion based on the statistics in 2014, indicating that older drivers account for only 7% of injured drivers but for 20% of driver deaths. Thus, older drivers’ fragility demands safer vehicles and transportation infrastructure with more effective protection in case of collisions. However, improving safety for older drivers should not depend only on external methods, which would be costly by the required upgrades to vehicles and infrastructure.

Improving the behavior and psychology of older drivers can be an alternative to improve safety if we can relate ageing to dangerous driving behaviors and stress. Various studies, including those mentioned above, have confirmed that older drivers have high death rates in cashes. However, as most crashes involve two or more road users, the responsibility of older drivers for accidents should be analyzed. Based on fatal crash data in the USA from 1999 to 2003, Tefft [17] found that the oldest drivers (above 85 years old) pose more risk to other road users than middle-aged drivers. In addition, Langford et al. [18] analyzed the insurance data from the state of Tasmania in Australia and found that older drivers (above 65 years old) were 1.5 times more likely to be responsible for crashes than middle-aged drivers. Likewise, Oxley et al. [19] summarized a series of studies and concluded that older road users were primarily responsible for crashes involving them. Thus, the study of older drivers’ behavior and psychology can be extremely important for improving road safety.

Before reporting the methods and findings from existing studies, we should define some important concepts:

1. Older driver. According to the Centers for Disease Control and Prevention (CDC) in the USA [20], an older driver is a license holder who is 65 years old or above. In contrast, the United Nations (UN) [3] define elder as people who are 60 years old or above. Moreover, the age that defines an older driver varies according to the country and organization, as further detailed in Section 3.1.
2. Driving stress. The UN [21] defines stress as “the physical and psychological process of reacting to and coping with events or situations that place extraordinary pressure upon a human being. Such events are usually sudden and often involve physical or emotional loss, such as witnessing casualties or destruction from combat or disasters or the serious injury or death of a relative, friend or co-worker.” Accordingly, driving stress is caused by negative factors in driving environment, such as traffic jam, being followed closely, and long-distance driving [22,23]. Meanwhile, functional abilities of drivers may also affect their driving stress. For example, a visually cluttered environments will increase driver’s stress if he/she is suffering from eyesight problems [24,25,26,27].
3. Driving behavior. Considering the meanings of drive and behavior from the Cambridge Dictionary [28,29], driving behavior can be defined as a set of actions taken by a driver to control a vehicle.
4. Dangerous driving behavior. A dangerous driving behavior is a driving behavior that can directly or indirectly cause a crash, injury, or death on the road [30,31,32]. It is also called risky driving behavior or bad driving behavior [33,34].

In the remainder of this paper, we provide a survey about several studies on the relationship between aging and driving behavior or stress. These studies often report apparently contradictory statements. For instance, European Commission [35] claimed that “an increase in age does not cause higher crash rates per exposure, challenging the traditional concept of a direct association between age-related deterioration of safety-relevant driving skills and driving performance.” Although the studies mentioned in this report present sound statistics and results, the findings contradict the facts and conclusions that we mention in the paragraphs above. However, various studies may be limited by their methods, as most of them relied on questionnaire surveys, whereas few considered driving simulators, and on-road experiments were rarely conducted. The inconsistent methods may result in conflicting conclusions. In fact, by analyzing the introduction and conclusions of the corresponding papers, we found that previous hypotheses were either confirmed or denied only by asking different participant groups to report their driving status in many cases. Although questionnaire surveys can provide reliable results, this method seems to repeatedly induce conflicting conclusions. On the other hand, studies based on driving simulations or on-road experiments have their own strengths and weaknesses, as discussed in detail in Section 3 and Section 4. As noted by van Wee and Banister [36] on their reviews, we do not aim to criticize one type of conclusions or completely rebut a method. Instead, we expect to address three questions in this review:

1. What approaches and conclusions were used and deduced while evaluating older people’s driving behavior and stress?
2. What are the advantages, disadvantages, and reliabilities of these approaches?
3. Is it possible to apply novel methods to study older drivers’ behavior and stress?

In Section 2, we describe the methodology adopted for preparing this review. Then, we present an overview on different methods and results regarding older adults’ driving behavior and stress, and state the advantages and disadvantages of existing methods in Section 3. In addition, we suggest a more comprehensive hybrid method to study older adults’ driving behavior and stress in Section 4. Finally, we draw conclusions in Section 5.

## 2. Methodology

We used ScienceDirect, Google Scholar, Taylor & Francis Online, Scopus, Web of Science, and less often CiNii (in Japanese) and CNKI (in Chinese) to search for papers reporting studies in fields related to older adults’ driving behavior and stress. To collect policy information and official reports, we mainly relied on two search engines: Google and Yahoo. The search terms included two groups: (1) old, older, older driver, older adult, and similar terms; (2) driving behavior, driving stress, and similar terms. In addition, driving behaviors were replaced by specific keywords, such as speeding, acceleration, and left/right turn. Logic OR was used within the same group, and logic AND was used across groups. Previous reviews were also searched, collected, and analyzed [37,38,39,40]. We reviewed more than 200 relative papers and reports. For introducing and comparing the latest studies and conclusions, about 120 references are included in this manuscript, and only about ten of them were published in the last century. Moreover, most sources, including official statistics and government information (e.g., population, population rates, policies, regulations), were no earlier than 2013.

## 3. Studies on Older Drivers’ Behavior and Stress: Objectives, Methods, and Results

### 3.1. Definition of ‘Older Driver’

Aging is a natural worldwide phenomenon that is being increasingly investigated in different areas. However, a basic but mostly overlooked aspect is the definition of ‘older people’, which can vary across organizations, countries, and studies. These varying definitions may lead to different conclusions. Moreover, disagreements and debates may originate and increase around different concepts, especially if they share the same name [41].

Table 1 lists definitions of ‘older people/drivers’ adopted by different organizations and countries. In general, 60 and 65 years old are the main age thresholds that determine whether an individual is considered as an elder, but no consensus has been achieved among official definitions.

In addition, the meaning of ‘older driver’ sometimes varies depending on various conditions even in the same country. This definition may reflect cultural differences and current situations in different countries. Japan is a typical example of this phenomenon. According to data from the World Bank [42], Japan is facing the most serious problem of population ageing worldwide, and thus has the highest threshold in all countries. Another important aspect is in the subdivision of older people/drivers. The European Union and UN define younger and older groups of people/drivers. Although these subdivisions are different, we found that the age group definition is important, not only among older adults, but also among individuals in other age groups. Some examples of age thresholds and the group subdivisions are listed in Table 2 and Table 3.

**Table 1:** Definition of ‘older people/driver’ according to different organizations and countries.

Organization/Country	Definition of Older People/Driver	Reference
European Union	Above 65 years old (Younger group: 65–74 years old Older group: 75 years old and above)	[8,43]
UN	Above 60 years old (Younger group: 60–79 years old Older group: 80 years old and above)	[3]
Australia	Depends on source (60 or 65 years old and above, or 60 years old and above divided into age groups spanning 10 years)	[44]
China	60 or 65 years old and above Driver over 70 years old must accept physical examination every year	[45,46]
France	65 years old and above has letter S (senior) in vehicle sticker	[47]
Germany	60 years old and above (but retirement age is 65 years)	[48]
Japan	65 years old and above (by Ministry of Internal Affairs and Communications statistics) 70 years old and above has vehicle sticker indicating senior 80 years old and above by the office of the Prime Minister of Japan	[49,50,51]
UK	65 years old and above	[9]
USA	65 years old and above	[20,52]

### 3.2. Data Usage, Collection and Analysis

Table 2 summarized four main kinds of data, which are mainly used for studies on older people’s driving behavior and stress.

By comparing data among different participants and driving conditions, researchers draw conclusions about older people’s driving and travel characteristics, and then give suggestions for driving safety.

The advantages and disadvantages of the different evaluation methods are discussed in Section 3.5.

### 3.3. Results about Older Drivers’ Behavior

Various studies [73,74,75,76,77] about driving behavior provide a gradual construction of a hierarchical system, in which behavior has different meanings for different transport users. Besides older driver, driving behavior should be defined in each study. In this review, we consider both driving behaviors (e.g., accelerating, decelerating, speeding, braking time/distance, turning time) and travel behaviors (e.g., road selection, left/right turn selection, trip frequency, trip length, non-home-based trip rate) as driving behaviors. Each indicator can evaluate the participant’s behavior as good or bad compared with a baseline, or as better or worse across groups. For instance, speeding can be measured directly during an experiment if the speed limit is known. In contrast, most indicators are relative and should be compared across age groups. In Section 3.4, common driving behavior indicators are listed along with biomedical indicators.

Two main and opposing results of studies on older drivers’ behavior can be summarized as follows:

1. Driving behaviors remain stable throughout the lifespan.
2. Driving behaviors deteriorate with age.

The gap between these two viewpoints has deepened enough to lead different research directions and vehicle/transportation design. Table 3 lists representative studies, in which 1/3 studies supported point 1 above. Although these papers do not represent the proportion of studies reaching to each result, we found that most studies determined that the driving behavior of older people is worse than that of people in other age groups.

As noted in Section 3.1, the definition of ‘older driver’ and comparison groups vary across studies. Although the studies seem to be focused on the same topic, we determined different study objects in most cases. For instance, 36-years-old and 75-years-old participants might belong to different age groups although some researchers defined both of them as “older drivers” [78,79,80,81]. Another example is that Delhomme et al. [82] divided participants into older and senior drivers groups but some other studies classified the participants into younger and middle-age groups [83,84]. Whether their conclusions relied on the classification method is still unclear [85].

From another viewpoint, Table 3 shows that most existing studies were based on self-reported information, and few considered experiments to collect on-road/in-vehicle data. It is difficult to determine the underlying reason for these study setups. We assume that questionnaire surveys are more appealing because they allow to gather much more data in a relatively shorter period compared to conducting experiments. However, each method has its own strengths and weaknesses. Despite its widespread use, the superiority of self-reported data over other methods is not conclusive. For example, several studies have reported that questionnaire and naturalistic driving data reached to the same conclusions only in a few situations (e.g., the habit of avoiding nighttime driving) [86,87,88]. Moreover, questionnaires may be more unreliable when applied to older drivers, possibly distorting conclusions. In Section 3.5, we discuss each method in more detail.

It is worth noting other conditions related to driving behaviors. Hakamies-Blomqvist and Wahlström [89] suggested that age–behavior relations must consider other factors such as gender. One of specific examples is that female older participants drove more carefully than male older drivers [90]. Some other researchers [91,92] also claimed that driving experience and living habits were real impact factors on driving and travel behaviors rather than age. For instance, retirement allows older adults to travel on weekdays and avoid rush hours. Therefore, driving behaviors such as speeding and road selection vary according to these specific factors. Shen et al. [93] confirmed that older adults are more likely to travel in the daytime, while younger adults drive more frequently after 16:00. Considering that fatal accidents in the daytime are only one-third of those at night [94], less road traffic incidents may be related to older drivers. Overall, results related to older drivers’ behavior suggest that although age is an important factor, it should be considered in the context of other factors.

**Table 3:** Methods and results from studies on older drivers’ behavior.

Result	Driving Behaviors Remain Stable throughout Lifespan				Driving Behaviors Deteriorate with Age			
Method	Reference	Age Defining Older Driver (Years)	Comparison Group(s)	Sample Size	Reference	Age defining Older Driver	Comparison Group(s)	Sample Size
Questionnaire, telephone, or official statistic	[42]	≥75	None	888	[54]	≥60	None	104
	[95]	≥55	18–34 years old, 35–54 years old	18–34 years old: 1522, 35–54 years old: 2726, >55 years old: 1883	[96]	Unknown (average age of 71.24)	Unknown(average age of 21.29 years)	Younger group: 24, Older group: 21.
					[82]	45–59: Older drivers, ≥60: senior drivers	18–29 years old, 30–44 years old	18–29 years old: 419, 30–44 years old: 357, 45–59 years old: 328, ≥60: 139
					[97]	≥55	None	1279
					[78]	36–60 years old (older group)	18–35 years old	216
					[98]	≥65	None	350
					[99]	–	–	–
Driving simulator	[84]	≥60	<25 years old: younger drivers *, 30–45 years old: middle-aged drivers	Younger drivers: 10, Middle-aged drivers: 11, Older drivers: 10	[100]	≥65	19–22 years old	Younger drivers: 12, Older drivers: 12
					[101]	≥60	18–40 years old	Younger drivers: 38, Older drivers: 38
					[102]	≥65	None	99
					[103]	≥65	18–33 years old (younger adults)	Younger adults: 45, Older adults: 40
					[104]	≥65	None	297
On-road/in-vehicle experiments	[79]	≥75	None	344				
	[80]	≥75	None	182				
	[83]	≥65	<25 years old: young drivers, 25–65 years old: middle-aged drivers	Unknown				

*: The group name is the same as that in the original reference.

### 3.4. Results about Older Drivers’ Stress

Stress has been proved to be a key factor in driving/transportation safety. Nearly fifty years ago, researchers claimed that stressful drivers were five times more likely to cause fatal accidents than others [105]. At the beginning of this section, we should note that stress is thought of as a result when coping resources might be insufficient to meet situational demands [106]. Thus, stress may be generated or escalated by not only age but also many subjective and objective factors [106,107,108,109].

Table 4 lists some representative factors that may influence driving stress.

Table 5 lists information from some representative studies on driving stress of older adults. Their research methods and conclusions, like the reviewed studies on driving behavior, have the following four characteristics:

1. The definition of ‘older driver’ either varies when comparing multiple age groups/single age group or is not established.
2. Most studies employed questionnaire surveys, and few considered on-road experiments.
3. Questionnaire surveys allowed collecting more data than studies based on simulators and on-road experiments.
4. Most studies concluded that driving stress increases with age, and few found significant differences between older adults and individuals in other age groups.

It is worth mentioning various results. Matthews et al. [106] evaluated the mechanism linking driving stress and behavior under various traffic situations. According to Dorn [107], although a stress–behavior relation exists, it is difficult to establish a fixed relation across age groups. Qu et al. [108] confirmed a correlation between driving stress and dangerous driving behaviors and claimed that gender should be considered as an interaction factor. These studies support the idea that age should not be evaluated as a single influencing factor when studying driving stress.

Unlike driving behavior, driving stress is difficult to measure directly. Lee and Winston [109] were clear: as the psychological reaction to driving situations often leads to dangerous driving behaviors, they can allow to evaluate driver’s stress. Therefore, there are two methods to determine driving stress through questionnaire surveys, either directly asking drivers to evaluate driving stress by themselves (i.e., driving stress questionnaire) or estimating the stress level from their driving behaviors. The second method is also adopted when using driving simulators or on-road experiments. Moreover, Lee and Winston [109] summarized stress-related driving behaviors and quantified stress–behavior correlations for estimation in other driving stress experiments. However, they also noted that the results are affected by participant’s emotional status, which is highly subjective and varies according to different factors, such as age, gender, and driving experience.

Stress is usually evaluated using physiological data. Although bio-signal collection and analysis technology have substantially evolved in the past decades, few studies on older adults’ driving stress have used this technology. This might be partially caused by the cost, because the same system setup should be used in each participant and in the same periods considering the influence of factors such as the circadian rhythm [116]. On the other hand, constructing relations among aging, stress, and physiological data may be difficult [117].

Table 6 lists common behavior indicators and biomedical indicators related to driving stress, where the latter can be measured by the corresponding medical devices.

### 3.5. Advantages and Disadvantages of Methods Related to Older Drivers’ Behavior and Stress

The methods we discuss in this section are related to data collection rather than analysis. As analysis includes tools such as regression models, significance tests, and other statistical methods, it should be discussed in mathematical contexts.

Studies based on questionnaire surveys allow obtaining a relatively large amount of data with reduced cost and collection time compared to other methods, so most researchers perform studies based on these surveys to evaluate older drivers’ behavior and stress. However, Ross et al. found that 3/4 older participants inclined to overrate their driving abilities in their study [98]. Therefore, the concealment and bias [118] are the disadvantages of questionnaire surveys.

The advantages and disadvantages of research using driving simulators somehow represent the midpoint between research using questionnaire surveys and on-road experiments. However, simulators can only reconstruct a simplified driving environment. The complexity of on-road environment may sometimes be even higher than that of flying environments because car drivers must face continuous interactions with on-road objects. In addition, the simulator environment may result unfamiliar to participants, possibly increasing the participants’ driving stress. Therefore, the participants’ behavior in a simulation experiment can differ from that in a real scenario [67,119].

On-road/in-vehicle experiments need pre-education for participants but are more subjective than self-report questionnaire. In addition, Vhaduri et al. [120] developed a GPS-stress model (GStress) to estimate driving stress and obtained better results than survey-based studies.

Biomedical data are also as subjective as on-road/in-vehicle data, but the participants are usually fewer than for questionnaire-based studies because of the high cost of biomedical sensors. Table 7 describes these factors for different data collection methods. Overall, researchers must consider a balance between these factors according to the expected results and available resources.

## 4. Improving Studies on Older Drivers’ Behavior and Stress

After comparing various methods to study older drivers’ behavior and stress, we can conclude that no single method can provide all the suitable experimental factors listed in Table 5. Alternatively, a possible improvement may be a hybrid method, in which researchers conduct two or more experiments in a study to leverage the advantages and compensate for the deficiencies of different methods.

Ross et al. [98] conducted a questionnaire survey study and driving simulator experiments. Kanamori et al. [117] collected questionnaire answers, on-road data, and biomedical information, and jointly analyzed the obtained information. Yokoyama and Takahashi [121] evaluated both objective and subjective stress indicators in the same experiment and established their mathematical relation. These researchers claim that a hybrid method increases the accuracy of results. Moreover, Ross et al. [98] found that simulator-based driving training before paper-based learning can significantly improve older adults’ driving abilities.

While evaluating older people’s driving behaviors and stress, we suggest an hybrid method:

First, researchers should collect participants’ subjective data by questionnaires. In this step, Manchester Driver Behavior Questionnaire, Driving Habits Questionnaire, and Driving Behavior Rating Scale [55,56,57,58,59,60] can be used. In second step, simulator, on-road, or both-based experiments can be conducted to obtain objective data, and then the relationship between the previous two kinds of data will be established. It should be noted that biomedical data is especially important in this method because it can evaluate the reliability of relation model. Finally, reflex and reaction responses should be considered when analyzing driving behaviors. As some behaviors involve interaction between two or more parties, the actions of the all the parties should be recorded and considered. To the best of our knowledge, no study has jointly considered questionnaire, simulator, and on-road/in-vehicle camera data to date, possibly due to the high costs, intense work, and high complexity associated with the experimental setup and data processing. Nevertheless, we expect that the continuously increasing computational capabilities may enable researchers to choose on-road/in-vehicle video data for more comprehensive analyses. Moreover, recent optimized methods and technological progress may lead to better tools or systems for both experiments and analyses.

## 5. Conclusions

By reviewing academic papers and government reports related to older drivers’ behavior and stress, we can answer the three questions posed in the introduction. First, questionnaire, driving simulator, and on-road/in-vehicle data have been used to collect driving information in different studies. Biomedical data collection can be added to simulator and on-road/in-vehicle experiments to further evaluate driving stress. Researchers have either compared older drivers and drivers in other age groups directly or subdivided participants into multiple age groups. Most existing studies and reports have varying definitions of ‘older driver’. In different studies, the age threshold in years for older driver can be 60, 65, or even higher. Consequently, the diverse grouping methods may influence study conclusions. Regarding driving behavior, some studies have reported deteriorating driving abilities with ageing, whereas others have not found significant differences between older drivers and drivers in other age groups. Two main conclusions have been drawn about driving stress: (1) driving stress escalates with age and (2) driving stress remains stable throughout the lifespan. It is worth noting that gender, driving experience, living positions, and some other factors should be considered while establishing regression model.

We found that most studies have been based on self-reported data, possibly due to its advantage of obtaining a relatively large dataset with reduced costs and in a short period. However, self-reported data lack objectivity, especially when collected from older drivers. In driving stress research, biomedical data can increase the reliability of results when collected from simulators or on-road experiments.

Until a new research model or technological system becomes available, we suggest using a hybrid method, which has already been used in several studies. This method requires collection of both subjective and objective data, and their relation is obtained through mathematical models. Moreover, on-road/in-vehicle cameras should be used to consider the action of other transport users, who usually influence driving behaviors. Based on data and analytical results, older drivers’ behavior and stress can be evaluated or estimated more comprehensively.

The contributions from this literature review can be summarizes as follows.

1. The review sums up methods and results from research relating older drivers, driving behaviors, and driving stress. It may provide guidelines to other researchers on the dimension and approach to conduct studies in this field.
2. It identifies problems in the objectives and methods adopted in existing research. For example, we cannot easily affirm or refute the conclusions of other studies, because their objectives may be different despite using similar terminology and concepts.
3. It suggests a hybrid method to mitigate bias in findings. Researchers can conduct their studies more objectively by applying surveys and collecting on-road/in-vehicle data. Biomedical indicators require further research to evaluate driving stress more objectively.

Overall, incident data have shown that older drivers are responsible for most crashes involving two or more road users, and some studies concluded that older drivers show risky driving behaviors. However, we should not only focus on accident rate because “traffic conflict” and “accident” represent different dimensions while evaluation driving behavior. As noted by Risser [122], a driver’s accident history may not reflect the performance of his driving behavior. Similarly, even a bad driver can stay accident-free for a relatively long period. Risser’s study measured several driving behaviors. Based on his research, we know that we should not easily say one driver had “good” or “bad” behavior without clarifying what elements of behavior were considered.

Moreover, we should note that not only driving education but also the underlying traffic system and car design should be improved, considering older drivers. For example, intelligent intersection infrastructures and advanced driver-assistance systems may reduce older drivers’ incidents in the future. Policy makers should not simply enforce older people to avoid driving, because the social and economic costs (e.g., supply of goods and services) will remain very high until the fundamental problems of older drivers’ safety are solved.

Last but not least, we urge the researchers in the field of older drivers’ behavior and psychology take time to reexamine their methodologies and conclusions because of the varying definitions of older driver and the objectivity of methods/sources. New findings or even opposite conclusions may be obtained in some research.

## Figures and Tables

**Table 2:** Four main data for evaluating older people’s driving behavior and stress.

Data Source	Brief Introduction	Representative Example(s)	Reference
Self-reported data	Collected from questionaire surveys or telephone interviews.	Manchester Driver Behavior Questionnaire Driving Habits Questionnaire Driving Behavior Rating Scale	[53,54,55,56,57,58,59,60]
Driving simulator data	Obtained from virtual driving environments.	Daimler’s new full-scale, high-dynamic driving simulator	[61,62,63,64,65,66]
On-road/in-vehicle data	Obtained from a variety of sensors, such as CAN (Controller are networks), cameras, and GPS.	Telematics box (T-box) GStress model	[67]
Biomedical data	Detected by biomedical sensors	Skin conductance response sensor Heart rate sensor RR interval sensor	[68,69,70,71,72]

**Table 4:** Influence factors on driving stress.

Category	Influence Factors On Driving Stress
Subjective Factors of Drivers	Interpersonal and job problems Overalll assessments of recent exposure to stressful life events Lack of driving knowledge, psychomotor skills and inexperience Depression after being involved in an accident (Post Traumatic Stress Disorder) Gender Dislike of driving Working condition (At-work drivers reported higher stress)
Objective Factors of Driving Environment	Increasing risk for driver accidents. e.g., traffic flow with high speed Close distance to other car(s) Driving when other stressed driver(s) is nearby

**Table 5:** Methods and results from studies on older drivers’ stress.

Result	Driving Stress Remains Stable throughout Lifespan				Driving Stress Increases with Age			
Method	Reference	Age Defining Older Driver (Years)	Comparison Group(s)	Sample Size	Reference	Age Defining Older Driver (Years)	Comparison Group(s)	Sample Size
Questionnaire, telephone interview, or official statistic	[110]	>34	Others	Older drivers: 130, Others: 165	[111]	≥67	48–67 years old: baby boomer	Baby boomer: 198, Older: 201
					[89]	≥70	None	Unknown
					[112]	Unknown	Unknown	914
					[113]	≥65	≤24 years old, 25–64 years old	≤24 years old: 109 25–64 years old: 423 ≥65 years old: 83
Simulator	[84]	>60	<25 years old: young drivers *, 30–45 years old: middle-aged drivers	Young drivers: 10, Middle-aged drivers: 11, Older drivers: 10	[102]	≥64	None	99
					[114]	≥65	18–25 years old: young	Young: 10, Old: 10
					[103]	≥65	18–33 years old: younger adults	Younger adults: 45, Older adults: 40
On-road/in-vehicle experiment					[115] **	≥65	≤25 years old: younger, 35–55 years old: middle-age	Younger: 20, Middle-age: 20, Older: 14

*: The group name is the same as that in the original reference. **: Used biomedical data.

**Table 6:** Behavior indicators and biomedical indicators related to driving stress.

Type		Indicator	Performance, Trend or Response under Stress
Driving behavior (general definition)	Driving behavior	Speed	Unstable and frequently changing between low and high values
		Speeding	Driver fails to control speed by unawareness of speeding
		Acceleration/deceleration	Sudden
		Braking time/distance	Sudden braking with shorter distance by late reaction to dangerous objects/conditions
		Turning time	Takes longer than without stress at intersections
		Lane-keeping	Inability to maintain the vehicle in lane center and frequent steering to adjust vehicle position
	Travel behavior	Road selection	Avoid expressways
		Left/right turn selection	Avoid turning right in countries where driving occurs on the left side of the road and vice versa
		Trip frequency	Avoid or give up driving
		Driving area and destination distribution	Prefer driving in familiar areas such as places near home
		Trip length	Avoid long-distance driving
		Driving time	Avoid driving at night, commuting times, and weekends
		Non-home-based trip rate	Low non-home-based trip rate when driving with stress and preference to home-based trips.
Biomedical data	Skin potential	Skin conductance response	Increase
		Skin conductance level	Increase
		Skin potential response	Increase
	Cardiovascular system	Diastolic blood pressure	Increase
		Heart rate	Increase
		Heart rate variability	Decrease
		Low-frequency component of heart rate variability	Increase
		Low-to-high frequency component ratio of heart rate variability	Increase
		RR interval	Decrease
		Systolic blood pressure	Increase
	Cerebrovascular system	Oxyhemoglobin	Increase
		Deoxyhemoglobin	Decrease
	Respiration	Respiration interval	Decrease
		Respiration rate	Increase
		Peripheral capillary oxygen saturation	Decrease
	Temperature	Skin temperature	Decrease

**Table 7:** Factors influencing methods to study older drivers’ behavior and stress.

Method/Source	Cost	Experiment Period	Sample Size	Objectivity
Questionnaire, telephone, or official statistic	+++	+++	+++	+
Driving simulator data	++	++	+	++
On-road/in-vehicle experiment data	++	+	++	+++
Biomedical data	+	++		+++

Markers +++, ++, and + indicate the factor rating from best to worst. Marker—indicates undefined rating. The experiment period and sample size for Biomedical data collection depend on the experimental method (simulator or on-road/in-vehicle experiment).

## Data Availability

Not applicable.
